# A radiomics-based nomogram for preoperative T staging prediction of rectal cancer

**DOI:** 10.1007/s00261-021-03137-1

**Published:** 2021-06-03

**Authors:** Xue Lin, Sheng Zhao, Huijie Jiang, Fucang Jia, Guisheng Wang, Baochun He, Hao Jiang, Xiao Ma, Jinping Li, Zhongxing Shi

**Affiliations:** 1 Department of Radiology, The Second Affiliated Hospital of Harbin Medical University, Harbin, China; 2 Research Lab for Medical Imaging and Digital Surgery, Shenzhen Institute of Advanced Technology, Chinese Academy of Sciences, Shenzhen, China; 3 Department of Radiology, the Third medical centre, Chinese PLA General Hospital, Beijing, China; 4 Department of Interventional Radiology, The Second Affiliated Hospital of Harbin Medical University, Harbin, China

**Keywords:** Nomogram, Rectal cancer, Staging, Magnetic resonance imaging

## Abstract

**Purpose:**

To investigate the value of a radiomics-based nomogram in predicting preoperative T staging of rectal cancer.

**Methods:**

A total of 268 eligible rectal cancer patients from August 2012 to December 2018 were enrolled and allocated into two datasets: training (*n* = 188) and validation datasets (*n* = 80). Another set of 32 patients from January 2019 to July 2019 was included in a prospective analysis. Pretreatment T2-weighted images were used to radiomics features extraction. Feature selection and radiomics score (Rad-score) construction were performed through a least absolute shrinkage and selection operator regression analysis. The nomogram, which included Rad-scores and clinical factors, was built using multivariate logistic regression. Discrimination, calibration, and clinical utility were used to evaluate the performance of the nomogram.

**Results:**

The Rad-score containing nine selected features was significantly related to T staging. Patients who had locally advanced rectal cancer (LARC) generally had higher Rad-scores than patients with early-stage rectal cancer. The nomogram incorporated Rad-scores and carcinoembryonic antigen levels and showed good discrimination, with an area under the curve (AUC) of 0.882 (95% confidence interval [CI] 0.835–0.930) in the training dataset and 0.846 (95% CI 0.757–0.936) in the validation dataset. The calibration curves confirmed high goodness of fit, and the decision curve analysis revealed the clinical value. A prospective analysis demonstrated that the AUC of the nomogram to predict LARC was 0.859 (95% CI 0.730–0.987).

**Conclusion:**

A radiomics-based nomogram is a novel method for predicting LARC and can provide support in clinical decision making.

**Supplementary Information:**

The online version contains supplementary material available at 10.1007/s00261-021-03137-1.

## Introduction

Colorectal cancer (CRC) is the most common tumor in the digestive system, and its mortality rate ranks third among cancer-related mortality in the world [1]. Moreover, rectal cancer accounts for about one-third of all CRC cases [2]. The primary treatments for rectal cancer include chemotherapy, radiotherapy, and surgery, though treatment options are determined by tumor stage. Early-stage rectal cancer can be treated directly by surgery, whereas locally advanced rectal cancer (LARC) requires neoadjuvant radiochemotherapy before surgery. Accurate preoperative staging of rectal cancer is essential for achieving precision treatment. Thus, it is vital to be able to precisely identify preoperative staging.

Magnetic resonance imaging (MRI) provides good soft-tissue contrast. The National Comprehensive Cancer Network [3] and the European Society for Medical Oncology [4] recommend MRI as the preferred imaging exam for rectal cancer. At present, MRI has been widely used in the evaluation of preoperative T stages of rectal cancer. However, it is not without limitations. Conventional MRI staging is easily influenced by clinical experience and the individual perspective of observers and its reproducibility and accuracy remains unsatisfactory [5–7]. In addition, conventional MRI has difficulty in distinguishing the inflammatory reaction around the tumor and tumor invasion, resulting in staging wrong [8]. Accurate staging is an imperative prerequisite to individualized therapy. The stratification according to accurate staging could avoid unnecessary chemoradiotherapy and their side effects, such as toxicity of chemotherapeutics, radiation enteritis, and rectal wall fibrosis and can reduce the financial burden of patients. Accurate staging has particular significance for clinical practice. Thus, there is a critical need to develop a method to provide this important information.

Radiomics can extract large amounts of quantified features from medical imaging data to provide mineable high-dimensional data. It deeply analyzes the clinicopathological information contained in large amounts of data [9, 10] and has been applied to tumor staging [11–13], predicting treatment response [14–17], and assessing the efficacy after chemoradiotherapy [18–20] in rectal cancer patients. Although some studies have explored the application of radiomics in T staging of rectal cancer, most of these studies used radiomics features alone; few have incorporated the clinical factors with the radiomics features and lack preoperative experimental validation. In this study, we introduced clinical factors and incorporated with radiomics features, analyzed from different aspects, constructed a multi-scale nomogram model to predict T staging in patients with rectal cancer, and validated the model in a prospective cohort, with the goal of providing a meaningful predictor and supporting for the individualized treatment plan, so as to make patients get more benefit from treatment.

## Materials and methods

### Patients

**Fig. 1:**
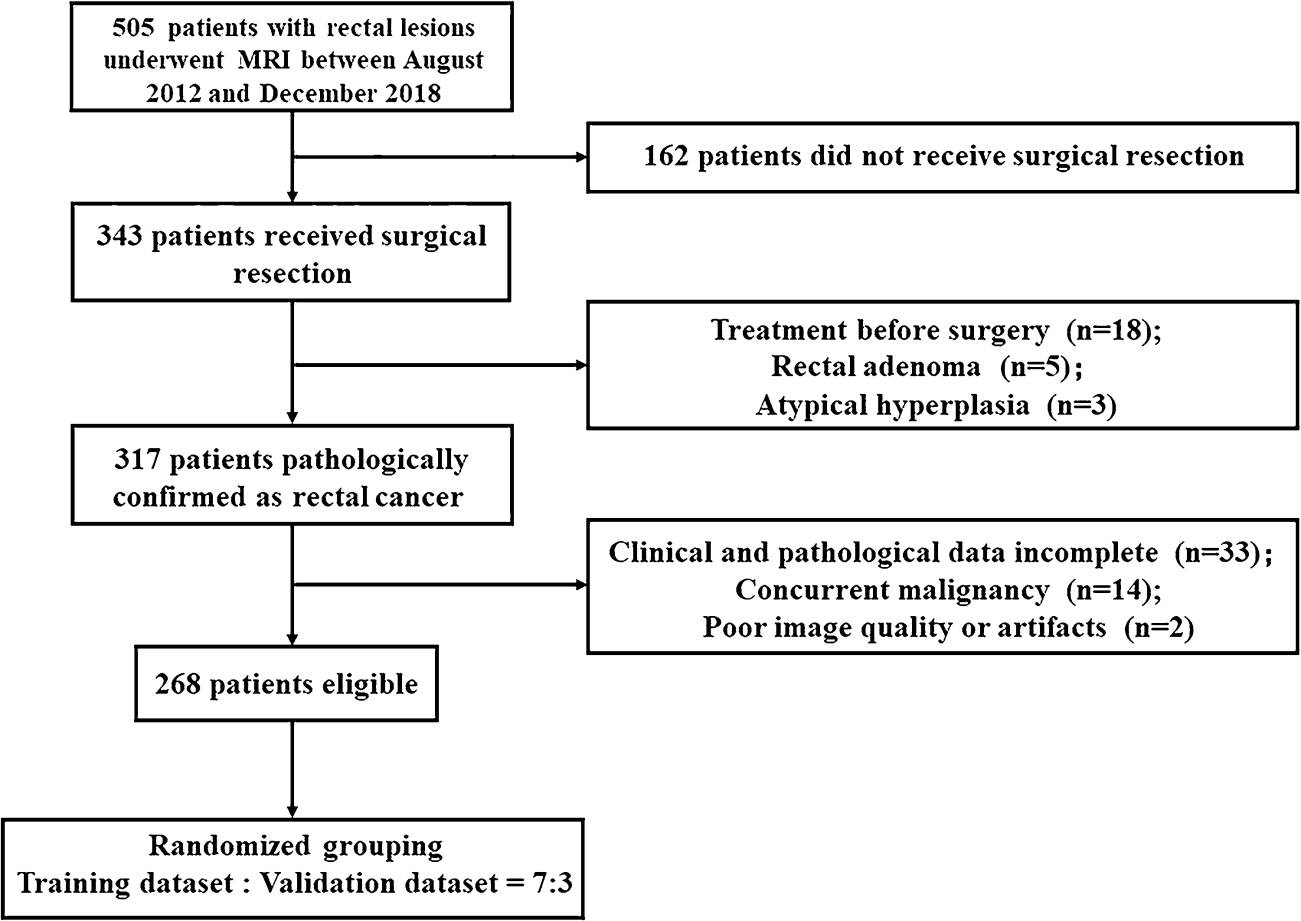
Patient selection process

We searched our retrospective database for consecutive patients who received rectal MRI examinations in our hospital from August 2012 to December 2018. Inclusion criteria were patients who (1) underwent surgery and pathologically diagnosed with rectal adenocarcinoma; (2) received no treatment before surgery; (3) underwent MRI within 2 weeks before surgery; and (4) had complete clinical and pathological data. Exclusion criteria were as follows: (1) preoperative therapy (radiotherapy, chemotherapy or chemoradiotherapy); (2) simultaneous existence of other malignant tumors; and (3) poor image quality or artifacts. Finally, 268 patients were eligible and randomly distributed into two datasets at a proportion of 7:3. The study was approved by the Ethics Committee and the need to obtain informed consent was waived. Figure 1 presents the patient recruitment process.

Baseline clinicopathologic data, including age, gender, preoperative carbohydrate antigen 199 (CA199), carcinoembryonic antigen (CEA), tumor diameter, location (distance from the anal verge), differentiation, and postoperative T staging, were derived from medical records.

### Pathologic evaluation

The specimens were fixed in formalin for 48 h, and two pathologists evaluated the tissue sections stained with hematoxylin–eosin (H–E). The pathological diagnosis was performed according to the 8th edition of the American Joint Committee on Cancer staging standard [21]. T1 tumors invade submucosa, T2 tumors invade muscularis propria, T3 tumors penetrate the muscularis propria to reach the subserosal layer or invade the adjacent rectal tissue without peritoneal covering, and T4 tumors penetrate the serosal membrane or directly invade other organs or tissues. T1–T2 stages were classified as early stage and T3–T4 stages as local advanced stage.

### MRI image acquisition and regions of interest segmentation

**Fig. 2:**
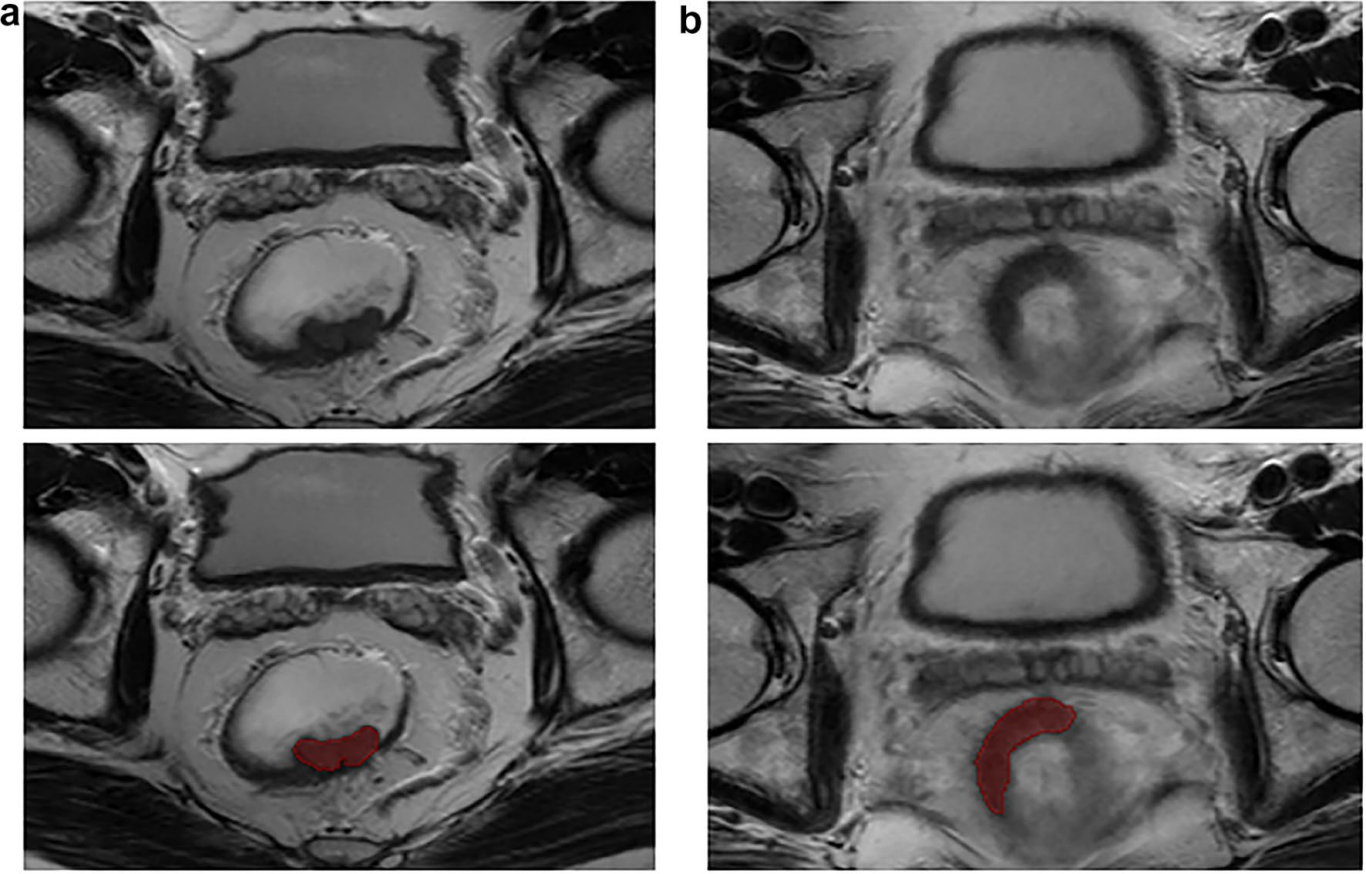
Tumor segmentation on rectal MRI. **a** A 49-year-old man with early-stage rectal cancer. **b** A 73-year-old man with LARC

Imaging data were collected using a GE Discovery MR750w 3.0 T MRI scanner with the phased-array body coil. Patients fasted for 4 h and emptied the bowel contents before scanning. The axial and coronal MRI sequences were perpendicular and parallel to the long axis of the rectal tumor, respectively. The regions of interest were manually outlined using each slice of the axial T2-weighted images to cover the entire tumor (Fig. 2a and b). Intestinal contents and air were excluded. The procedure was performed using the MITK software (MITK Workbench 2018.04.2, http://mitk.org/wiki/MITK). Thirty cases of MRI images were randomly selected, and two radiologists with 9 (doctor 1) and 11 years (doctor 2) of MRI interpretation experience independently outlined the tumors without knowing the pathological results. Doctor 1 repeated the process 1 week later. The intra-class correlation coefficient (ICC) was used to assess the inter-/intra-observer variability. An ICC above 0.75 was considered to have good reproducibility. Doctor 1 finished the delineation for the remaining images. The formulation of ICC is shown in Supplementary Material 1.

### Radiomics feature extraction

Pyradiomics [22] (versions 3.0, https://www.radiomics.io/pyradiomics.html) were used to extract radiomics features. For the original MRI images, a Laplacian of Gaussian (LoG) filter with different σ parameters and a wavelet filter were used for preprocessing to obtain filtered images. Then original and filtered images were taken to extract features. Each patient obtained 960 features from their images and these features were normalized using a *Z*-score transformation. Supplementary Material 2 lists the features.

### Feature selection and radiomics signature building

After extracting features, a least absolute shrinkage and selection operator (LASSO) logistic regression was performed to identify the optimal predictive features from the training dataset. Then the optimal features weighted by corresponding coefficients were linearly combined to obtain a radiomics score (Rad-score) [23].

### Radiomics nomogram construction and performance evaluation

The Rad-score and clinicopathological factors, including age, gender, tumor location, maximum diameter, differentiation, CEA, and CA199, were considered as possible predictive factors. Significant predictors were chosen via univariate and multivariate logistic regression of the training dataset. Based on Akaike’s information criterion, we used the likelihood ratio test to make a backward stepwise selection. A radiomics nomogram grounded on the multivariate logistic analysis was built for the training dataset.

The predictive ability of the nomogram was quantified through the area under the curve (AUC) of a receiver operator characteristic (ROC) curve. A calibration curve [24] was chosen to evaluate the calibration performance via bootstrapping with 1000 resamplings. The Hosmer–Lemeshow (H–L) test was used to evaluate goodness of fit of the nomogram.

### Validation of the nomogram

In the validation dataset, each patient received a Rad-score and the performance of the nomogram was validated. The discrimination was evaluated by the AUC, and calibration was verified by the calibration curve and H–L test.

### Clinical use

The clinical utility of the nomogram was assessed via quantifying the net benefits at different thresholds in decision curve analysis (DCA) [25] for the training and validation datasets.

### Statistical analysis

A Mann–Whitney U test or an independent samples *t*-test was used for continuous variables comparison, while Chi-squared tests for categorical variables. R software (version 3.6.3; https://www.r-project.org/) was applied to statistical analysis. *P* < 0.05 was considered statistically significant. Supplementary Material 3 describes the packages used in R.

## Results

### Patient characteristics

The median age was 59.84 in the training dataset and 60.20 in the validation dataset. Males had a preponderance in both datasets (69.7% and 70.0%, respectively). The moderately differentiated patients accounted large proportion in two datasets (70.2% and 70.0%, respectively). No significant differences were found among clinical factors between the two datasets. The training dataset contained 133 LARC patients and the validation dataset contained 60 LARC patients. There was no difference in the proportion of LARC patients in the two datasets (Table 1).

**Table 1:** Characteristics of patients

Clinical factors	Training dataset (*n* = 188)	Validation dataset (*n* = 80)	*P*
Age, mean ± SD, years	59.84 ± 10.77	60.20 ± 10.67	0.799
Gender, no (%)			0.958
Male	131(69.7)	56(70.0)	
Female	57(30.3)	24(30.0)	
Tumor diameter, median (IQR), cm	4.5(3.5–5.5)	4.5(3.6–5.5)	0.428
Location			0.339
Lower	65(34.6)	21(26.3)	
Middle	119(63.3)	56(70.0)	
Upper	4(2.1)	3(3.7)	
Differentiation degree			0.940
Poorly	21(11.2)	10(12.5)	
Moderately	132(70.2)	56(70.0)	
Well	35(18.6)	14(17.5)	
CEA level, no (%)			0.329
Normal	120(63.8)	46(57.5)	
Abnormal	68(36.2)	34(42.5)	
CA199 level, no (%)			0.080
Normal	162(86.2)	62(77.5)	
Abnormal	26(13.8)	18(22.5)	
Stage, no (%)			0.478
T1–T2	55(29.3)	20(25.0)	
T3–T4	133(70.7)	60(75.0)	

*SD* standard deviation, *IQR* interquartile range, *CEA* carcinoembryonic antigen, *CA199* carbohydrate antigen 199

### MRI evaluation of T staging

**Fig. 3:**
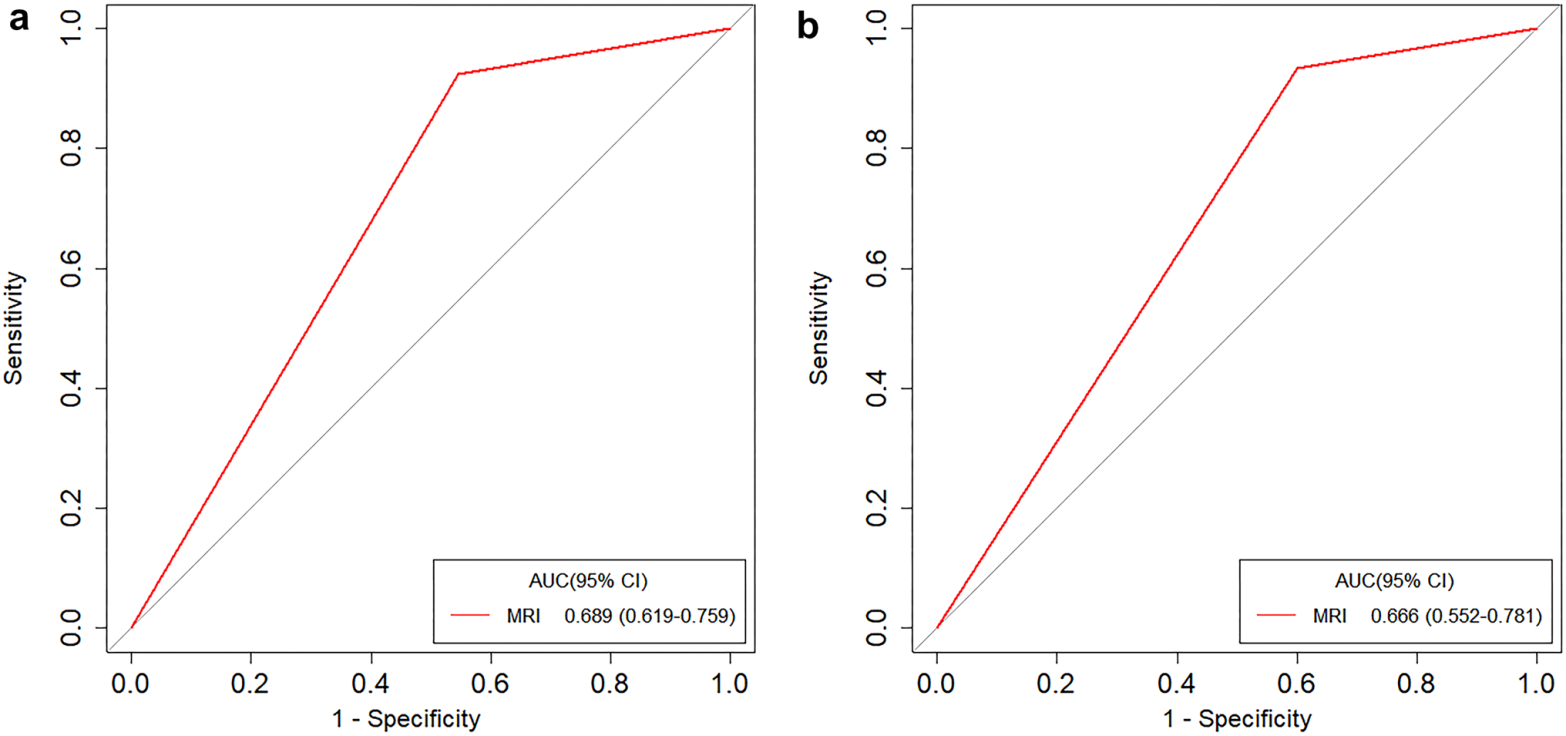
The ROC of the training (**a**) and validation (**b**) datasets for the MRI

Two radiologists evaluated the T staging through MRI images without knowing the pathological results. When they had different opinions, the final results were determined through discussion. The AUC was used to evaluate the performance of the radiologists' subjective judgment of T staging through MRI images. The AUC for the training dataset was 0.689 (95% CI 0.619–0.759) and 0.666 (95% CI 0.552–0.781) for the validation dataset (Fig. 3a and b).

### Repeatability of ROI segmentation, feature selection, and radiomics signature construction

**Fig. 4:**
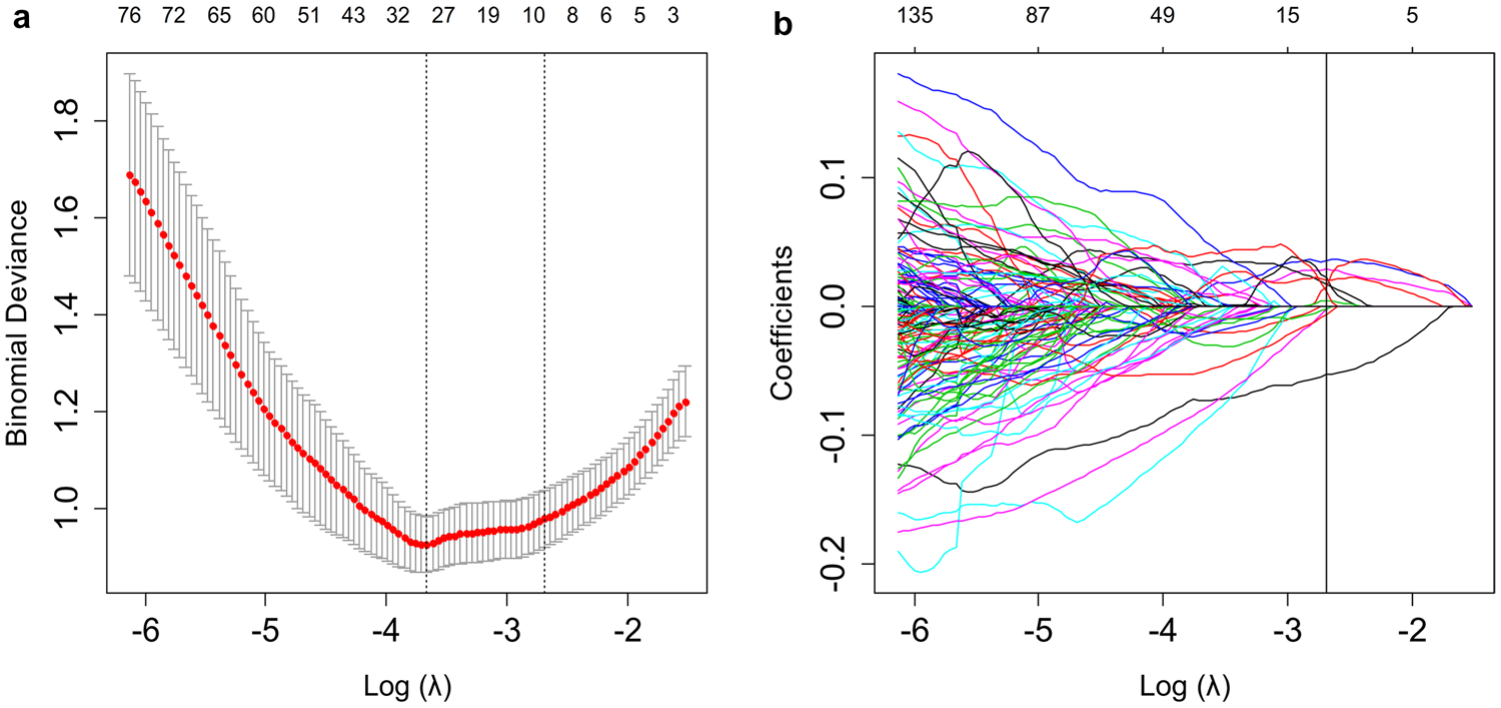
The LASSO logistic regression model for radiomics feature selection. **a** The tuning parameter (*λ*) selection was based on a tenfold cross-validation in the LASSO model. The minimum criteria and one standard error of the minimum criteria (1-SE criteria) were used to place the vertical lines at the optimum values. A lambda of 0.068, with log(*λ*) = − 2.688 was chosen (1-SE criteria) for selecting features. **b** The LASSO coefficient profiles. The value selected by cross-validation was applied for drawing the vertical line and nine nonzero coefficients were shown

The evaluation of intra-/inter-observer repeatability of ROI segmentation was through calculating ICC. The intra- and inter-observer ICC were 0.816–0.947 and 0.778–0.925, respectively, which indicated satisfying repeatability of the segmentation. In the training dataset, the LASSO logistic regression identified nine potential radiomics features with nonzero coefficients (Fig. 4a and b). There were three shape-based features, one LoG filter feature and five wavelet filter features. In order to explore the contribution of the shape-based features, we built two Rad-scores: Rad-score 1 consisted of all nine features and Rad-score 2 consisted of the remaining features after removing the shape-based features. Then the AUC was used to evaluate the performance of Rad-scores. The AUC of Rad-score 1 was 0.872 (95% CI 0.821–0.922) for the training dataset and 0.807 (95% CI 0.705–0.909) for the validation dataset. The AUC of Rad-score 2 was 0.867 (95% CI 0.815–0.919) for the training dataset and 0.786 (95% CI 0.671–0.901) for the validation dataset. The performance of Rad-score 1 was better than Rad-score 2, and we chose Rad-score 1 as the final Rad-score. The Rad-score calculation formula was as follows:

Rad-score = 1.087604 + 0.090569 × log-sigma-5–0-mm-3D_firstorder_Kurtosis + 0.204631 × original_shape_Maximum 2D Diameter Row – 0.222923 × original_shape_Sphericity + 0.116812 × original_shape_Minor Axis Length + 0.140837 × wavelet-HLL_glcm_Difference Entropy + 0.379834 × wavelet-HLL_glszm_Size Zone NonUniformity + 0.017470 × wavelet-HLH_glszm_Zone Entropy – 0.041454 × wavelet-HHL_firstorder_Mean + 0.004327 × wavelet-LLL_glcm_Informational Measure of Correlation 1

The Rad-scores of LARC patients were generally higher than those who had early-stage rectal cancer. A significant difference was found between LARC and early-stage rectal cancer Rad-scores (mean ± standard deviation) in both the training dataset (1.402 ± 0.868 vs 0.208 ± 0.680, respectively, *P* < 0.001) and the validation dataset (1.476 ± 0.836 vs 0.488 ± 0.782, respectively, *P* < 0.001).

### Individualized radiomics nomogram development and validation

**Fig. 5:**
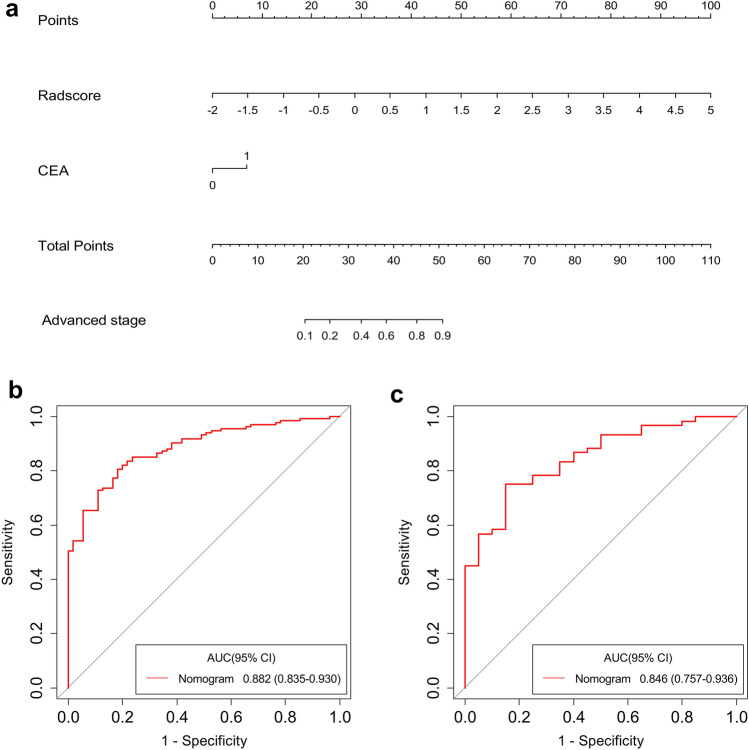
Radiomics nomogram for predicting LARC. The nomogram combining CEA and Rad-score was built from the training dataset (**a**). The ROC curves of the training (**b**) and validation (**c**) datasets for the radiomics nomogram

Univariate logistic regression analysis indicated that Rad-score, tumor diameter, tumor location, CEA, and CA199 level had predictive value for LARC. Further multivariate logistic regression analysis confirmed CEA level and Rad-score as independent predictive factors (Table 2). Thus, we developed a nomogram that combined Rad-score and CEA (Fig. 5a). The AUC of the nomogram was 0.882 (95% CI 0.835–0.930) for the training dataset and 0.846 (95% CI 0.757–0.936) for the validation dataset (Fig. 5b and c).

**Table 2:** Risk factors for patients with LARC

Factors	Univariate logistic regression		Multivariate logistic regression	
	OR (95% CI)	*P* value	OR (95% CI)	*P* value
Age	1.01 (0.98–1.04)	0.484	Not selected	Not selected
Gender	1.40 (0.69–2.84)	0.352	Not selected	Not selected
Tumor diameter	2.14 (1.60–2.87)	** < 0.001**	0.99 (0.68–1.43)	0.937
Location	2.02 (1.09–3.75)	**0.026**	1.47 (0.67–3.22)	0.339
Differentiation degree	0.71 (0.39–1.27)	0.249	Not selected	Not selected
CEA level	4.08 (1.85–9.00)	** < 0.001**	2.68 (1.03–7.00)	**0.044**
CA199 level	5.83 (1.33–25.62)	**0.019**	1.95 (0.39–9.66)	0.416
Rad-score	8.41 (4.31–16.38)	** < 0.001**	7.45 (3.12–17.75)	** < 0.001**

*P* values with statistical significance are shown in bold

*LARC* locally advanced rectal cancer, *OR* odds ratio, *CI* confidence interval, *CEA* carcinoembryonic antigen, *CA199* carbohydrate antigen 199

**Fig. 6:**
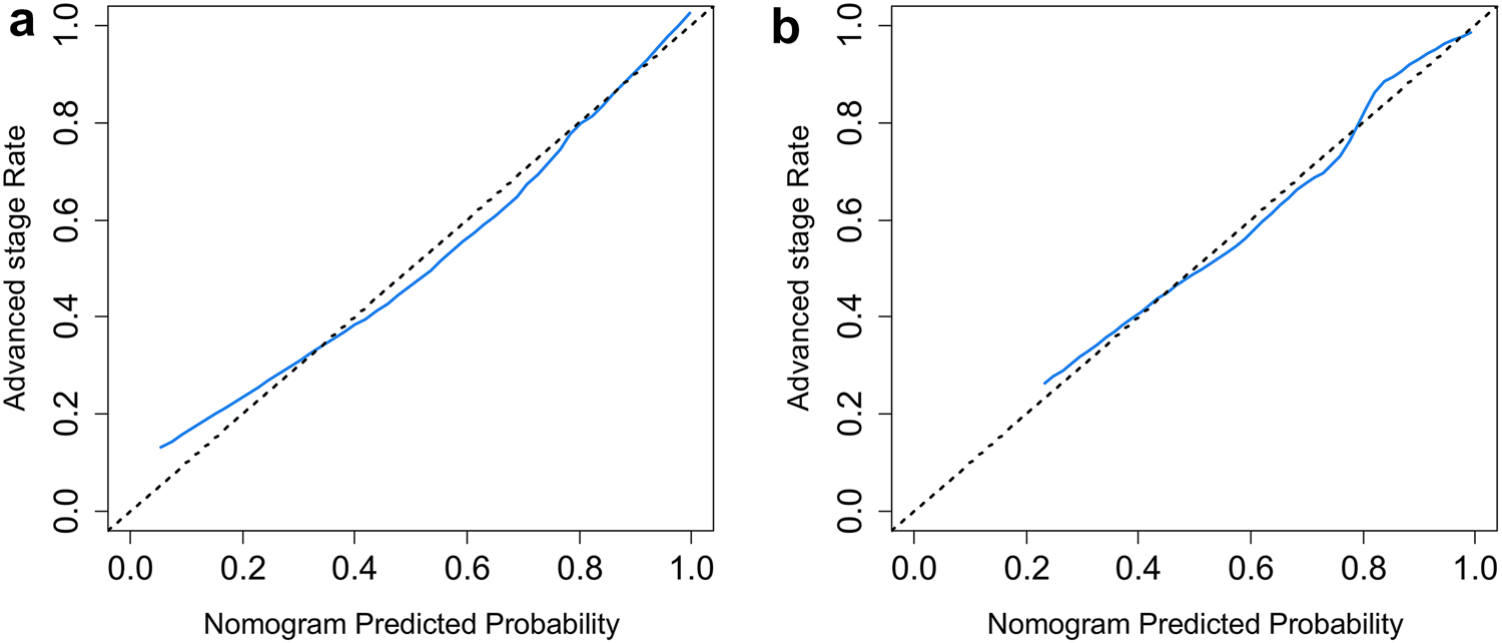
The calibration curves of the training (**a**) and validation (**b**) datasets

The calibration curve (Fig. 6a and b) and the H–L test results (*P* = 0.768 for the training dataset and 0.638 for the validation dataset) indicated good consistency between observation and prediction.

### Clinical utility

**Fig. 7:**
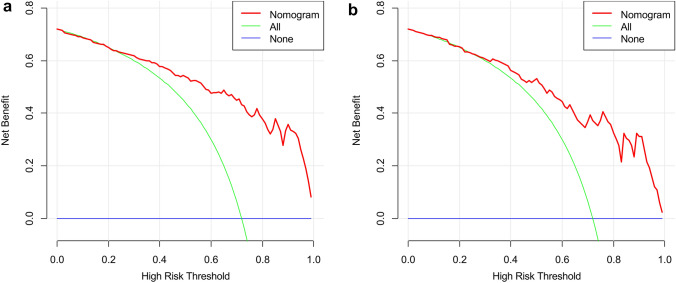
The DCA of the training (**a**) and validation (**b**) datasets

The DCA for radiomics nomogram indicated that when the probability of LARC was between 0.23–0.99 and 0.27–0.99 in the training (Fig. 7a) and validation datasets (Fig. 7b), respectively, using the nomogram to evaluate pathological LARC had added benefit compared with treating all patients as early or local advanced stage.

### Prospective trial analysis

The radiomics nomogram was used to perform prospective analysis on another 32 patients with rectal cancer, including 18 males and 14 females. Table 3 presents the patient characteristics.

**Table 3:** Characteristics of patients in the prospective study

Clinical factors	Prospective study
Age, mean ± SD, years	63.59 ± 10.04
Gender, no (%)	
Male	18(56.2)
Female	14(43.8)
Tumor diameter, median (IQR), cm	4.5(4.0–5.5)
Location	
Lower	14(43.8)
Middle	17(53.1)
Upper	1(3.1)
Differentiation degree	
Poorly	3(9.4)
Moderately	26(81.2)
Well	3(9.4)
CEA level, no (%)	
Normal	23(71.9)
Abnormal	9(28.1)
CA199 level, no (%)	
Normal	26(81.2)
Abnormal	6(18.8)
Stage, no (%)	
T1–T2	10(31.3)
T3–T4	22(68.7)

*SD* standard deviation, *IQR* interquartile range, *CEA* carcinoembryonic antigen, *CA199* carbohydrate antigen 199

**Fig. 8:**
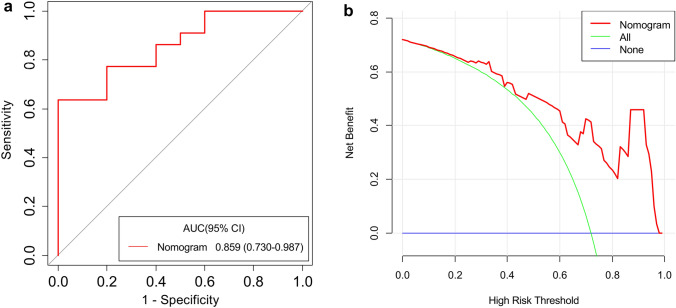
The ROC (**a**) and DCA (**b**) of the nomogram

The AUC of the radiomics nomogram was 0.859 (95% CI 0.730–0.987) (Fig. 8a). The DCA showed that when the probability of LARC ranged from 0.06 to 0.97 (Fig. 8b), using the nomogram to evaluate pathological LARC had added benefit compared with treating all patients as early or local advanced stage, which suggested the nomogram had good clinical utility. The prospective analysis showed that this nomogram has the satisfying predictive ability in prospective conditions.

## Discussion

In our study, we explored the performance of a radiomics-based nomogram for the preoperative individual prediction of T stage in rectal cancer patients. The nomogram incorporated radiomics features and CEA levels. The results showed that this nomogram had good accuracy and clinical utility in both the retrospective analysis and prospective study. This indicated that radiomics-based nomograms can improve preoperative T stage prediction strategies in rectal cancer patients, which has important implications for clinical decision making.

Computed tomography (CT), MRI, and positron emission tomography/CT are commonly used imaging methods in clinical routine work. MRI is the preferred imaging exam for rectal cancer T staging [3, 4]; however, there are some drawbacks that limit its application in preoperative assessment. Conventional MRI has difficulty in distinguishing tumor infiltration from fibrosis [26]. Most importantly, the accuracy of conventional MRI in the diagnosis of rectal cancer T staging is influenced by subjectivity [27].

Radiomics is a promising field that focuses on enormous quantitative information extraction from medical images and deeply explore the potential connections related to tumor occurrence and development [28, 29]. It can provide evidence to support clinical decision making and thus has high clinical significance [14, 19, 30]. In this study, we constructed and validated a radiomics nomogram, which combined both the radiomics signature and clinicopathologic risk factors for personal prediction of T staging in rectal cancer patients. We used axial T2-weighted images to extract features of the entire tumor and the independent predictors of LARC were selected out by LASSO logistic method. This method allows us to combine radiomics features into a radiomics signature [31–33]. Multi-factor analysis that incorporates individual factors into a factor panel has been widely used in recent studies [34–36]. For example, Wang et al. [34] constructed an MRI-based radiomics model to predict the muscle-invasive status of bladder cancer and confirmed that the radiomics could be an efficient tool for preoperative prediction. Similarly, Xu et al. [35] developed a radiomics nomogram to predict intracerebral hematoma expansion and found that the nomogram could serve as a convenient measurement. Pan et al. [36] used the LASSO logistic method to identify optimal radiomics features for preoperative classification of ovarian cystadenoma. The results showed that imaging biomarkers could classify ovarian serous cystadenoma and mucinous cystadenoma. In our study, we incorporated the Rad-score and CEA levels into a nomogram for preoperative prediction of LARC. The nomogram exhibited good discrimination in retrospective cohort studies and was confirmed in the prospective pilot analysis as well. Furthermore, our nomogram performed better than previous studies. Ma et al. [11] used MRI radiomics derived from T2-weighted images to predict pathological characteristics of rectal cancer. They extracted 1029 radiomics features and used the LASSO method to select optimal features. Eleven features were found to be related to T staging and the accuracy of T stage prediction was 0.762. Yin et al. [12] collected data from 115 rectal cancer patients in their study. Texture features based on apparent diffusion coefficient (ADC) maps and diffusion-weighted images (DWI) were used to predict different stages of rectal cancer, which resulted in an AUC of 0.793. Finally, Sun et al. [13] explored the role of radiomics features in identifying tumor characteristics of rectal cancer. They demonstrated that it is feasible to use MRI-based radiomics features to predict T staging.

In our study, we constructed the nomogram based on the T2-weighted imaging, which is a routine sequence in rectal scanning. T2-weighted imaging has better stability in appearance. High-resolution T2-weighted imaging can provide clear visualization of rectal layers by offering good contrast between the tumor and surrounding tissue, making it one of the most important sequences for staging of rectal cancer [37, 38]. Contrast-enhanced T1-weighted images are obtained by scanning after intravenous injection of a contrast agent to determine angiogenesis inside tumors. However, for staging of rectal cancer, contrast-enhanced T1-weighted imaging is not recommended as a routine sequence [39]. In our study, we extracted features from original and filtered images, including wavelet filter and LoG filter images. The features derived from the wavelet filter images accounted for the majority of the final Rad-score. Wavelet filters have advantages in signal denoising and have been commonly applied in recent studies [40–42]. One prospective study that predicted tumor grading of rectal carcinoma had features obtained primarily from wavelet filter images in their radiomics signature [40]. In the study by Hamerla et al. [41], the most relevant features in their radiomics model for predicting pathological response were also extracted from wavelet filter images. Furthermore, Liang et al. [42] constructed a prediction model for metachronous liver metastases in rectal cancer patients, and a large proportion of the selected features was obtained from wavelet filter images.

In addition to radiomics features, we introduced CEA into the nomogram. It is one of the commonly used tumor markers in clinical practice [43]. CEA is a cell surface glycoprotein and is expressed at low levels in normal tissue but is up-regulated in most CRC lesions [44, 45]. Previous studies have demonstrated that CEA is correlated with rectal cancer staging [46–48]. In this study, we analyzed the performance of CEA in predicting LARC. After univariate and multivariate logistic regression, we found that CEA was a predictive factor for LARC, fitting the results of previous studies. The model constructed by combining CEA with a radiomics signature showed good accuracy and calibration ability.

Some limitations exist in this study. First, the patients recruited in the study were from a single center, and therefore, our study lacks external validation. Second, the sample size was relatively limited. Third, our images were all scanned by one 3.0 T MRI scanner, which may lead to a limitation in model generalization. In the future, multi-center, large-scale, and multi-vendor studies are needed to overcome these limitations.

In conclusion, we constructed a radiomics-based nomogram and validated its prediction ability and clinical utility. The nomogram is a novel method to predict LARC and can provide support for clinical decision making.

## Supplementary Information

Below is the link to the electronic supplementary material.

Supplementary file1 (PDF 187 kb)

## Data Availability

The datasets used and/or analyzed during the current study are available from the corresponding author on reasonable request.
