# Supplementary material for: A radiomics-based nomogram for preoperative T staging prediction of rectal cancer
Source: Abdom Radiol (NY). 2021 Jun 3;46(10):4525–35. doi: 10.1007/s00261-021-03137-1 (PMC8435521; doi:10.1007/s00261-021-03137-1)
Supplement: Supplementary file 1 — Supplementary file1 (PDF 187 kb) [file 261_2021_3137_MOESM1_ESM.pdf]

Supplementary Material

Article title: A radiomics-based nomogram for preoperative T staging prediction of rectal cancer

Journal name: Abdominal Radiology

Authors: Xue Lin[1,2], Sheng Zhao[1], Huijie Jiang[1*], Fucang Jia[2*], Guisheng Wang[3*], Baochun He[2], Hao Jiang[1], Xiao Ma[2], Jinping Li[1], Zhongxing Shi[4]

1. Department of Radiology, The Second Affiliated Hospital of Harbin Medical University, Harbin, China
2. Research Lab for Medical Imaging and Digital Surgery, Shenzhen Institutes of Advanced Technology, Chinese Academy of Sciences, Shenzhen, China
3. Department of Radiology, the Third medical centre, Chinese PLA General Hospital, Beijing, China.
4. Department of Interventional Radiology, The Second Affiliated Hospital of Harbin Medical University, Harbin, China

Corresponding authors: Huijie Jiang, E-mail: jhjemail@163.com; Fucang Jia, E-mail: fc.jia@siat.ac.cn; Guisheng Wang, E-mail: wanggs1996@tom.com

1. The formulation of ICC

The formulation of ICC we used was

$$ICC = \frac{\sigma_s^2}{\sigma_s^2 + \sigma_\epsilon^2}$$

where σ_s denotes variance caused by differences between the segmentations and σ_ϵ denotes variance caused by differences between the points in the segmentations [1].

2. Radiomics features

The parameters used for radiomics features extraction were as follows: bin-width = 5; normalize = true; resampled pixel spacing = [3, 3, 3]; sigmas used for LoG filter = 3.0 and 5.0; features were extracted in 3D. The extracted features were divided into six groups. Detailed descriptions can be viewed at https://pyradiomics.readthedocs.io/en/latest/features.html:

Group 1: Shape and size-based features

Group 2: First-order features

Group 3: Gray level co-occurrence matrix (GLCM) features

Group 4: Gray level run length matrix (GLRLM) features

Group 5: Gray level size zone matrix (GLSZM) features

Group 6: Gray level dependence matrix (GLDM) features

Group 1: Shape and size-based features (14 features)

1. Elongation
2. Flatness
3. Least Axis Length
4. Major Axis Length
5. Maximum 2D Diameter Column
6. Maximum 2D Diameter Row
7. Maximum 2D Diameter Slice
8. Maximum 3D Diameter
9. Mesh Volume
10. Minor Axis Length

11. Sphericity
12. Surface Area
13. Surface Volume Ratio
14. Voxel Volume

The above features were obtained from the original images.

Group 2: First-order features (198 features)

1. 10 Percentile
2. 90 Percentile
3. Energy
4. Entropy
5. Interquartile Range
6. Kurtosis
7. Maximum
8. Mean Absolute Deviation
9. Mean
10. Median
11. Minimum
12. Range
13. Robust Mean Absolute Deviation
14. Root Mean Squared
15. Skewness
16. Total Energy
17. Uniformity
18. Variance

The above features were obtained from the original images (18 features), Laplacian of Gaussian (LoG) filter (36 features) and wavelet filter (144 features) processed images.

Group 3: GLCM features (242 features)

1. Autocorrelation
2. Joint Average
3. Cluster Prominence
4. Cluster Shade
5. Cluster Tendency
6. Contrast
7. Correlation
8. Difference Average
9. Difference Entropy
10. Difference Variance
11. Joint Energy
12. Joint Entropy
13. Informal Measure of Correlation 1, IMC 1
14. Informal Measure of Correlation 2, IMC 2
15. Inverse Difference Moment, IDM
16. Inverse Difference Moment Normalized, IDMN
17. Inverse Difference, ID
18. Inverse Difference Normalized, IDN
19. Inverse Variance
20. Maximum Probability
21. Sum Entropy
22. Sum Squares

The above features were obtained from the original images (22 features), LoG filter (44 features) and wavelet filter (176 features) processed images.

Group 4: GLRLM features (176 features)

1. Gray level non-uniformity
2. Gray level non-uniformity normalized

3. Gray level variance
4. High gray level run emphasis
5. Long run emphasis
6. Long run high gray level emphasis
7. Long run low gray level emphasis
8. Low gray level run emphasis
9. Run entropy
10. Run length non-uniformity
11. Run length non-uniformity normalized
12. Run percentage
13. Run variance
14. Short run emphasis
15. Short run high gray level emphasis
16. Short run low gray level emphasis

The above features were obtained from the original images (16 features), LoG filter (32 features) and wavelet filter (128 features) processed images.

Group 5: GLSZM features (176 features)

1. Gray level non-uniformity
2. Gray level non-uniformity normalized
3. Gray level variance
4. High gray level zone emphasis
5. Large area emphasis
6. Large area high gray level emphasis
7. Large area low gray level emphasis
8. Low gray level zone emphasis
9. Size zone non-uniformity
10. Size zone non-uniformity normalized
11. Small area emphasis

12. Small area high gray level emphasis
13. Small area low gray level emphasis
14. Zone entropy
15. Zone percentage
16. Zone variance

The above features were obtained from the original images (16 features), LoG filter (32 features) and wavelet filter (128 features) processed images.

Group 6: GLDM features (154 features)

1. Dependence entropy
2. Dependence non-uniformity
3. Dependence non-uniformity normalized
4. Dependence variance
5. Gray level non-uniformity
6. Gray level variance
7. High gray level emphasis
8. Large dependence emphasis
9. Large dependence high gray level emphasis
10. Large dependence low gray level emphasis
11. Low gray level emphasis
12. Small dependence emphasis
13. Small dependence high gray level emphasis
14. Small dependence low gray level emphasis

The above features were obtained from the original images (14 features), LoG filter (28 features) and wavelet filter (112 features) processed images.

3. R packages used in statistical analysis

LASSO logistic regression was performed using the "glmnet" package. The AUC calculation and ROC curve plotting were conducted using the "pROC" package. Nomogram construction and calibration were based on the "rms" package. The H-L test

was conducted using the “generalhoslem” package. The DCA was carried out using the “rmda” package.

1. Taha AA, Hanbury A (2015) Metrics for evaluating 3D medical image segmentation: analysis, selection, and tool. BMC Med Imaging 15:29
